# Emergency Department Use of Genicular Nerve Block for Sickle Cell Pain Crisis: A Case Report

**DOI:** 10.5811/cpcem.65803

**Published:** 2026-10-26

**Authors:** Rosemary Beranek, Hayley Musial, Daniel Parsons-Moss, Andrea Dreyfuss

**Affiliations:** * Hennepin County Medical Center, Department of Emergency Medicine, Minneapolis, Minnesota; † Essentia Health, Department of Emergency Medicine, Duluth, Minnesota

**Keywords:** genicular nerve block, regional anesthesia, sickle cell disease, acute pain crisis, case report

## Abstract

**Introduction:**

Acute pain crises are a common emergency department presentation in patients with sickle cell disease. Pain management typically relies on multimodal analgesia, with opioids often playing a central role. We propose incorporating regional anesthesia techniques into the pain management regimen.

**Case Report:**

We present a case of a young patient with sickle cell disease experiencing an acute pain crisis with severe knee pain. A genicular nerve block was performed, resulting in significant pain reduction in the affected knee.

**Conclusion:**

Regional anesthesia, including isolated nerve blocks, may be safe and effective as part of a multimodal pain approach for patients suffering from acute pain crises due to sickle cell disease. This may help reduce the need for opioid or other sedating pain medications, as well as the inherent risks associated with increasing doses, such as respiratory depression.

## INTRODUCTION

One of the most common presentations of sickle cell disease (SCD) to the emergency department (ED) is sickle cell crisis or acute pain crises resulting from vaso-occlusion of vessels.^1^,^2^ The mainstay of treatment for these patients in the acute setting often involves the use of opioid medications.^3^ These medication regimens may limit the opioid doses patients can receive in the ED, frequently necessitating inpatient admission if pain control remains inadequate.

Although few data are available regarding the use of regional anesthesia in the ED for controlling chronic pain, the available data shows improvement in pain scores and decreased opiate use.^4^ To date, there are no cases in the emergency medicine literature documenting the use of regional anesthesia in the ED as part of multimodal pain control for SCD patients in acute pain crises. Most of the published research describing the use of regional anesthesia for SCD patients focuses on epidural anesthesia.^5^,^6^ To our knowledge, no case reports to date have described the use of genicular blocks for sickle cell-related pain. These blocks have been described in anesthesia literature following knee replacements or knee trauma but have not yet been adopted into widespread use in the ED.^7^–^10^

## CASE REPORT

We present the case of a 27-year-old male with SCD who presented to the ED with acute sickle cell pain, mainly in his right knee and wrist. The patient had a history of sickle cell beta thalassemia, osteonecrosis of the lumbar spine, and splenomegaly, with multiple prior presentations for vaso-occlusive crises managed with hydroxyurea, glutamine, and crizanlizumab infusions.

The patient called emergency medical services when his pain was unrelieved with his prescribed oxycodone. Paramedics administered 0.5 mg of intravenous (IV) hydromorphone. In the ED, he initially received 15 mg IV ketorolac, 975 mg oral acetaminophen, and 1 mg IV hydromorphone. Over the next several hours, the patient required several more doses of 0.5 to 1 mg IV hydromorphone boluses before he was eventually started on a patient-controlled analgesia pump of hydromorphone. He developed intermittent mild to moderate hypoxia due to decreased respiratory drive, requiring 3–4 liters of supplemental oxygen via nasal cannula. The patient was still uncomfortable, complaining of persistent knee pain despite the patient-controlled analgesia. The team proposed a nerve block to reduce this pain. He consented to a genicular block, which was performed at the bedside. The patient reported an improvement in pain from a “10/10 to a 2/10” approximately 30 minutes after the procedure. He was admitted to the hospital for ongoing management of his sickle cell crisis.

Sensory innervation of the knee is complex and involves terminal sensory branches of the femoral, obturator, and sciatic nerves. The anterior knee is divided into four quadrants, each with a respective genicular nerve branch: the superomedial genicular nerve, the superolateral genicular nerve, the inferomedial genicular nerve, and the inferolateral (ILGN) genicular nerve.

While the American Society of Regional Anesthesia and Pain Medicine recommends blocking three of these nerves but omitting the ILGN to prevent accidentally blocking the adjacent common peroneal nerve (which could result in foot drop),^5^ the physician may opt to target this nerve as well to provide greater pain coverage. Anesthesia guidelines also suggest blocking the femoral nerve branch innervating the vastus intermedius, which lies anteriorly along the femur, to provide anesthesia over the patellar region.

Our block targeted all four genicular nerves. The patient did not receive a block to the femoral nerve due to physician discretion and patient preference to forgo a fifth injection. The procedure took approximately 35 minutes. Ropivacaine 0.5% was used, which generally takes 10–20 minutes to take effect. The block may last up to 15 hours.

The sonographic landmarks for these nerves are relatively simple to find. The landmarks are shallow and require visualization of the bony cortex of the femur and tibia. With little overlying soft tissue, these landmarks are superficial; therefore, a linear probe is used (Image 1). The objective of the procedure is not to visualize the nerves themselves but rather the bony cortex at the slope between the shaft and epicondyles of the long bones. Color Doppler is an additional tool used to visualize the four genicular arteries, which run in the genicular neurovascular bundle. For the inferior landmarks, the tibial metaphysis is the target landmark (Images 2 and 3), whereas for the superior nerves it is the femoral epicondyle.

*CPC-EM Capsule*

What do we already know about this clinical entity?

*Nerve blocks are safe and effective for many causes of musculoskeletal pain or injuries as an adjunct to standard pain regimens.*

What makes this presentation of disease reportable?

*A genicular block relieved acute knee pain in a patient with sickle cell disease.*

What is the major learning point?

*Specific pain sites, such as joints, can be targeted for pain reduction using regional anesthesia.*

How might this improve emergency medicine practice?

*Incorporating regional anesthesia into a multimodal regimen offers targeted pain control and minimizes total analgesic use.*

## DISCUSSION

This case represents the first documented use of a genicular block for sickle cell-related pain in the emergency setting. For patients with acute pain crisis, management often requires repeated doses of opioids or patient-controlled anesthesia pumps. These large or stacked doses can cause significant respiratory depression, which greatly increases morbidity risk.^3^ Using multimodal analgesia to improve pain, while reducing opioid consumption, has become increasingly recognized as best practice in pain management.

Regional anesthesia has emerged as a valuable component of multimodal analgesia in emergency medicine. While few data exist specifically for regional anesthesia in sickle cell disease, growing evidence supports its use for acute pain management in the ED. A systematic review by Ritcey et al of the use of regional analgesia for hip and femoral neck fractures in the ED found that nerve blocks significantly reduced pain scores and opioid consumption compared to standard analgesia alone.^4^ While the review focused on traumatic injuries rather than chronic pain, it demonstrates the feasibility and efficacy of implementing regional anesthesia in the ED.

Beyond hip fractures, emergency physicians have successfully employed various peripheral nerve blocks for acute pain control. Burl et al demonstrated successful use of posterior tibial nerve blocks for plantar frostbite pain with improved pain scores.^11^ Additionally, Nagel et al described femoral and fascia iliaca blocks to manage proximal traumatic femur fractures. They also compared the safety of these peripheral nerve blocks to a case where use of opioids caused oversedation of a patient, which led to her being intubated.^12^ These studies collectively support the principle that targeted nerve blocks can provide effective analgesia while minimizing systemic medication side effects and complications.

Genicular nerve blocks have primarily been described in the chronic pain and anesthesia literature for managing knee osteoarthritis. Randomized controlled trials by Shanahan et al and Kim et al demonstrated the efficacy of ultrasound-guided genicular nerve blocks in decreasing pain scores in patients with chronic knee osteoarthritis. These studies established genicular blocks as viable options for chronic knee pain management in outpatient settings.^7^ This was also assessed by Kim et al in a randomized controlled trial to assess efficacy of genicular nerve blocks with anesthetic alone or in combination with a corticosteroid.^8^ While their study goal was to compare the differences in medication type, they documented success in pain reduction with the peripheral nerve block, regardless of whether corticosteroids were used.

More recently, genicular nerve blocks have been successfully applied in the ED for acute and subacute knee pain. Sobel and Oswald reported the first case of a 3-point genicular nerve block for acute traumatic knee pain in the ED, demonstrating rapid pain relief and improved mobility.^10^ Kong et al subsequently described successful management of subacute osteoarthritic knee pain in the ED using genicular blocks.^9^

The ultrasound-guided technique offers several advantages for emergency physicians. The anatomic landmarks—the bony cortex at the femoral epicondyles and tibial metaphysis—are superficial and relatively consistent across patients. Unlike many nerve blocks that require direct visualization of neural structures, the genicular block targets the nerves based on their consistent anatomic relationship to bony landmarks and associated vessels. This makes the learning curve more accessible for physicians developing their regional anesthesia skills.

The application of regional anesthesia to chronic pain populations, particularly patients with sickle cell disease, represents an underexplored frontier. Given that vaso-occlusive crises commonly affect specific joints and anatomic regions, targeted peripheral nerve blocks offer a logical approach to pain control.

## LIMITATIONS

There were multiple challenges that we faced. The patient reported pain in multiple joints, which made complete pain relief impossible. He had been placed on a patient-controlled hydromorphone pump prior to the genicular block. After the block, he began complaining of more pain in his wrist, requesting an additional block at that site. While his pain was mostly relieved in his knee, he did require ongoing IV parenteral medications for remaining pain in other joints. This is an expected limitation of regional anesthesia in SCD patients. It was also difficult to measure reduction in the patient’s use of patient-controlled anesthesia postblock for the same reasons. To learn more about the benefits of this block for pain crises in patients who do not yet require patient-controlled anesthesia, in.future we aim to offer the block to SCD patients earlier in their presentation.

There were also challenges to performing the procedure itself. Because this block requires five injection sites to successfully block the entire knee, multiple injection sites can be time-consuming for the physician and uncomfortable for the patient. However, with extensive practice and greater ease in finding the landmarks, this block has the potential to be a useful adjunct for pain relief, given the superficiality and relative anatomic consistency of the landmarks.

## CONCLUSION

This case report demonstrates a novel application of genicular nerve blocks as an adjunct in managing localized joint pain for sickle cell patients presenting to the ED in acute pain crisis. Although regional anesthesia has become more established in trauma settings, its use in chronic pain populations—particularly those with sickle cell disease—remains underexplored. This case supports the feasibility, safety, and efficacy of genicular nerve blocks in the ED, especially when standard opioid-based regimens are limited by side effects or insufficient relief. Further investigation is warranted to assess its broader utility, optimal timing, and impact on outcomes such as opioid consumption, hospital length of stay, and patient satisfaction. Using regional anesthesia may serve as a meaningful step toward multimodal pain control and improved care for a historically undertreated and vulnerable population.

## Figures and Tables

**Image 1:**
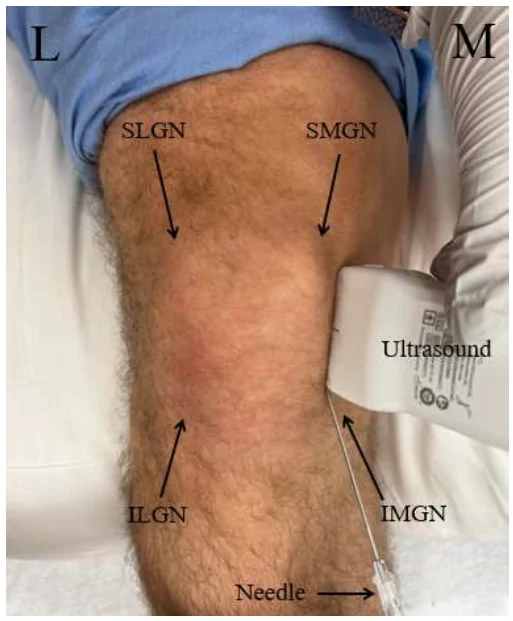
Four injection sites for genicular nerve block, with linear ultrasound probe and needle at the inferomedial genicular nerve site. In clockwise order from the probe are the inferolateral, superolateral, and superomedial nervie sites. *ILGN*, inferolateral genicular nerve; *IMGN*, inferomedial genicular nerve; *L*, lateral; *M*, medial; *SLGN*, superolateral genicular nerve

**Image 2:**
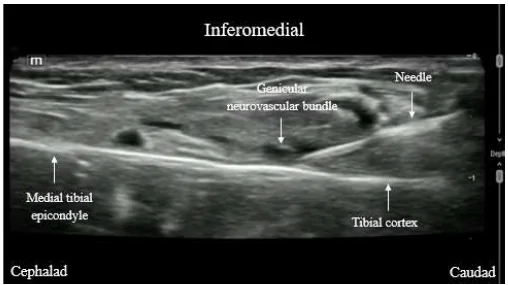
Ultrasound image with relevant anatomy labeled for the inferomedial genicular nerve and the needle visualized in-plane with bony target of the tibial cortex.

**Image 3:**
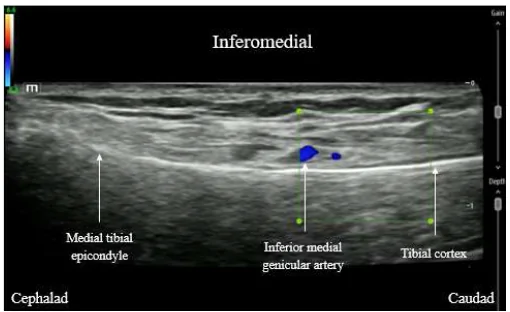
Ultrasound image with relevant anatomy labeled for the inferomedial genicular nerve, needle visualized in-plane with bony target of tibial cortex. Color Doppler identifies the artery in the genicular neurovascular bundle.
